# GnRH Neuron Excitability and Action Potential Properties Change with Development But Are Not Affected by Prenatal Androgen Exposure

**DOI:** 10.1523/ENEURO.0362-22.2022

**Published:** 2022-12-05

**Authors:** Jennifer Jaime, Suzanne M. Moenter

**Affiliations:** 1 The Neuroscience Graduate Program, University of Michigan, Ann Arbor, MI 48109; 2 Departments of Molecular and Integrative Physiology, University of Michigan, Ann Arbor, MI 48109; 3 Internal Medicine, University of Michigan, Ann Arbor, MI 48109; 4 Obstetrics and Gynecology, University of Michigan, Ann Arbor, MI 48109; 5 The Reproductive Sciences Program, University of Michigan, Ann Arbor, MI 48109

**Keywords:** development, electrophysiology, GnRH neurons, intrinsic properties, prenatal androgenization

## Abstract

Gonadotropin-releasing hormone (GnRH) neurons produce the final output from the brain to control pituitary gonadotropin secretion and thus regulate reproduction. Disruptions to gonadotropin secretion contribute to infertility, including polycystic ovary syndrome (PCOS) and idiopathic hypogonadotropic hypogonadism. PCOS is the leading cause of infertility in women and symptoms resembling PCOS are observed in girls at or near the time of pubertal onset, suggesting that alterations to the system likely occurred by that developmental period. Prenatally androgenized (PNA) female mice recapitulate many of the neuroendocrine phenotypes observed in PCOS, including altered time of puberty, disrupted reproductive cycles, increased circulating levels of testosterone, and altered gonadotropin secretion patterns. We tested the hypotheses that the intrinsic properties of GnRH neurons change with puberty and with PNA treatment. Whole-cell current-clamp recordings were made from GnRH neurons in brain slices from control and PNA females before puberty at three weeks of age and in adulthood to measure GnRH neuron excitability and action potential (AP) properties. GnRH neurons from adult females were more excitable and required less current to initiate action potential firing compared with three-week-old females. Further, the afterhyperpolarization (AHP) potential of the first spike was larger and its peak was delayed in adulthood. These results indicate development, not PNA, is a primary driver of changes to GnRH neuron intrinsic properties and suggest there may be developmentally-induced changes to voltage-gated ion channels in GnRH neurons that alter how these cells respond to synaptic input.

## Significance Statement

Gonadotropin-releasing hormone (GnRH) neurons play a crucial role in reproductive function. Disruptions to the release of pattern of GnRH secretion are implicated in fertility disorders, such as polycystic ovary syndrome (PCOS). Prenatally androgenized (PNA) female mice recapitulate many of the neuroendocrine phenotypes observed in women diagnosed with PCOS. We used electrophysiology to study how the intrinsic properties of GnRH neurons are altered with pubertal development and with PNA treatment. We found that prepubertal versus postpubertal GnRH neurons had different properties, including increased excitability after puberty. PNA treatment did not affect these typical developmental changes. These data suggest the postulate that development, rather than androgen exposure, is a primary regulator of the voltage-gated ion channels of GnRH neurons.

## Introduction

Gonadotropin-releasing hormone (GnRH) is the final output from the brain for the neuroendocrine control of reproduction. GnRH release occurs in an episodic manner from neurons located in the midventral preoptic area and hypothalamus (Clarke and Cummins, 1982; Moenter et al., 1992; Knobil and Neill, 1994). GnRH acts on the anterior pituitary to stimulate synthesis and release of the gonadotropins follicle-stimulating hormone (FSH) and luteinizing hormone (LH); low-frequency GnRH pulses favor these processes for FSH whereas high-frequency GnRH pulses favor LH (Wildt et al., 1981; Haisenleder et al., 1991). In females, FSH and LH regulate ovarian follicle maturation and steroidogenesis. Disruptions to gonadotropin secretion patterns, implying altered GnRH release, can contribute to infertility including idiopathic hypothalamic hypogonadism (Legro, 2003; Tsutsumi and Webster, 2009; Sidhoum et al., 2014) and polycystic ovary syndrome (PCOS; Legro, 2003; Burt Solorzano et al., 2012). PCOS is the leading cause of infertility in females, affecting up to 20% of females of reproductive age according to the Rotterdam criteria, for which patients must exhibit at least two of three symptoms: hyperandrogenemia, oligo/anovulation, and/or polycystic ovarian morphology (Chang and Katz, 1999; Legro, 2003; McCartney and Marshall, 2016). Hyperandrogenemic PCOS is diagnosed in 8–10% of all women and gonadotropin release in these women is characterized by persistently high LH pulse frequency and elevated LH/FSH ratio (Taylor et al., 1997; McCartney et al., 2002; Patel et al., 2004; Tsutsumi and Webster, 2009), suggesting at least some changes are occurring centrally at the level of GnRH release.

To study underlying central mechanisms, we must turn to animal models that recapitulate aspects of PCOS (Walters et al., 2018). In several species, including primates, mice, rats and sheep, *in utero* exposure to androgens produces offspring that develop reproductive neuroendocrine phenotypes similar to hyperandrogenemic PCOS (Levine et al., 1985; Robinson et al., 2002; Sullivan and Moenter, 2004; Foecking et al., 2005; Mahoney and Padmanabhan, 2010; Roland and Moenter, 2011; Witham et al., 2012; Moore et al., 2015; Abbott et al., 2016; Coyle and Campbell, 2019). In adulthood, prenatally androgenized (PNA) mice exhibit disrupted estrous cycles, elevated LH pulse frequency and increased testosterone levels compared with controls (Roland and Moenter, 2011; Moore et al., 2015; Dulka and Moenter, 2017; Berg et al., 2018). GnRH neurons from adult PNA mice have a higher action potential (AP) firing rate (Roland and Moenter, 2011; Dulka and Moenter, 2017). This elevated firing rate is likely driven at least in part by PNA-induced increases in GABAergic transmission to these cells (Sullivan and Moenter, 2004; Berg et al., 2018); unlike most neurons, GABA signaling via the GABA_A_ receptor can induce action potential firing by GnRH neurons (DeFazio et al., 2002; Herbison and Moenter, 2011). Whether or not intrinsic changes in GnRH neurons also contribute to the increased firing rate of these cells in adult PNA animals is not known.

The onset of PCOS is thought to occur near puberty (Ibañez et al., 1993; Franks, 2002; Rosenfield, 2007; Ibáñez et al., 2009; Burt Solorzano et al., 2010) and studies in PNA mice suggest neurobiological changes are already occurring before puberty is complete. Specifically, in contrast to the increased firing rate observed in GnRH neurons from PNA adults, GnRH neurons from three-week-old PNA mice have reduced firing rates compared with controls (Roland and Moenter, 2011; Dulka and Moenter, 2017). Interestingly, GnRH neurons from three-week-old PNA mice still receive increased GABAergic transmission compared with controls (Berg et al., 2018). The decrease in GnRH neuron activity in combination with the increase in GABA transmission to these cells in three-week-old PNA mice was surprising because we would expect the increase in GABA drive to increase firing activity unless the response to GABA is altered. Indeed, PNA treatment decreases the percentage of GnRH neurons from three-week-old PNA mice that increase firing in response to locally-applied GABA, suggesting their response to GABA is indeed altered. This was not attributable to changes in either baseline membrane potential or the reversal potential for GABA_A_-mediated current (Berg et al., 2018), indicating that either other elements of GABA_A_ receptor signaling, for example receptor composition, and/or changes in other intrinsic properties of GnRH neurons contribute to the reduced ability of GABA to induce action potential firing in GnRH neurons from prepubertal PNA mice.

Here, we began to characterize the response of GnRH neurons to current injection to examine how development and**/**or PNA treatment alter intrinsic properties of these cells. Specifically, we examined the excitability of GnRH neurons, defined as the number of action potentials generated as a function of current injected, and their response to hyperpolarizing current injections. By performing these studies in both control and PNA mice at three weeks of age and in adulthood, we gained valuable information about the normal development of GnRH neuron excitability during the pubertal process, and how PNA treatment affects this developmental trajectory. We hypothesized that GnRH neurons from PNA mice are less excitable at three weeks of age compared with controls, but become more excitable than controls during adulthood.

## Materials and Methods

All chemicals were acquired from Sigma-Aldrich unless noted.

### Animals

GnRH-GFP (Tg(Gnrh1-EGFP)51Sumo MGI:6158457) mice (Suter et al., 2000) were bred in our colony. All mice were provided with water and Harlan 2916 (nonbreeders) or 2919 (breeders) chow *ad libitum* and were held on a 14/10 h light/dark cycle with lights on at 3 A.M. Eastern Standard Time. To generate PNA mice, female GnRH-GFP transgenic mice on a C57Bl/6J background and a CD1 female were bred with a C57Bl/6J male and monitored daily for a copulatory plug (day 1 of pregnancy). The CD1 dam assists in providing maternal care and nutrition. On days 16–18 of pregnancy, GnRH-GFP dams were injected subcutaneously with 225 μg/d of dihydrotestosterone (DHT) for PNA or sesame oil for vehicle controls. Combined litter sizes were adjusted to <15 pups by culling CD1 pups to standardize nutrition. Adult female mice were studied on the morning of diestrus, determined via vaginal cytology and uterine mass. All procedures were approved by the Institutional Animal Care and Use Committee of the University of Michigan.

### Brain slice preparation

All solutions were bubbled with 95% O_2_/5% CO_2_ for at least 15 min before use with tissue and throughout the experimental recordings. Brain slices were prepared 4–8.5 h after lights on as described (DeFazio and Moenter, 2002). Brains were removed and placed in ice-cold sucrose saline containing the following (in mm): 250 sucrose, 3.5 KCl, 26 NaHCO_3_, 10 D-glucose, 1.25 Na_2_HPO_4_, 1.2 MgSO_4_, and 3.8 MgCl_2_ (350 mOsm). Coronal slices (300 μm) through the hypothalamic region were cut with a Leica VT1200S Microtome (Leica Biosystems). Slices were incubated for 30 min at room temperature (∼21–23°C) in 50% sucrose saline and 50% artificial CSF (ACSF; containing (in mm): 135 NaCl, 3.5 KCl, 26 NaHCO_3_, 10 D-glucose, 1.25 Na_2_HPO_4_, 1.2 MgSO_4_, and 2.5 CaCl_2_, 315 mOsm, pH 7.4). Slices were then transferred to 100% ACSF solution at room temperature for 0.5–5 h before recording. A minimum of five mice from at least four litters were studied per group; up to four recordings were used per mouse.

### Recording solutions and data acquisition

Whole-cell patch-clamp recordings in current-clamp mode were conducted to investigate the intrinsic properties of GnRH neurons from PNA and control mice before and after puberty. The pipet solution contained (in mm): 125 K gluconate, 20 KCl, 10 HEPES, 5 EGTA, 0.1 CaCl_2_, 4 MgATP, and 0.4 NaGTP, 305 mOsm, pH 7.2 with NaOH. This solution is based on the native intracellular chloride concentrations in GnRH neurons determined using gramicidin perforated-patch recordings (DeFazio et al., 2002; Berg et al., 2018). A 14.5-mV liquid junction potential was negated online before each recording. During recordings, brain slices were continuously perfused with carboxygenated ACSF (3 ml/min) and kept at 30–31°C with an inline-heating unit (Warner Instruments Model SH-27B). GFP-positive GnRH neurons were visualized with infrared differential interference contrast and fluorescence microscopy on an Olympus BX51WI microscope. All recordings were made using an EPC-10 patch-clamp amplifier and a computer running PatchMaster software (HEKA Elektronik). To monitor recording quality, input resistance, series resistance, baseline current, and capacitance were monitored throughout experiments from the averaged membrane current response to 16 hyperpolarizing voltage steps from –70 mV (5 mV, 20 ms, acquisition 100 kHz, filter 10 kHz). Data were analyzed using IgorPro (Wavemetrics). Only recordings with input resistance of >500 MΩ, stable compensated series resistance of <20 MΩ and a stable capacitance were used for analysis.

### Experimental design

GnRH neuron excitability, the number of action potentials generated as a function of current injection, and action potential properties were monitored in GnRH neurons in the preoptic area of brain slices from prepubertal (18–21 d) and adult (84–130 d) gonad-intact control and PNA female mice; adults were studied in diestrus, determined by vaginal cytology. PNA status was confirmed by monitoring age at vaginal opening (VO), estrous cyclicity by vaginal lavage for 14 consecutive days, and measuring anogenital distance (AGD) between 70 and 84 d of age (mean of three successive daily measures).

### Excitability and action potential property analysis

To characterize GnRH neuron excitability and action potential properties, current-clamp recordings were obtained (20-kHz acquisition, 10-kHz filter) in the presence of ionotropic glutamate and GABA receptor antagonists [20 μm D-APV (Tocris), 10 μm CNQX (Tocris), 100 μm picrotoxin]. Bridge balance (95%) was used for all experiments. Cells were maintained within 1.5 mV of –70 mV. Basal membrane potential was calculated during the 1 ms before the start of current injection. Current steps (500 ms, 5-pA increments from 0 to +40 pA, 5-pA decrements from 0 to –50 pA) were delivered to test the membrane potential response. The first action potential (AP) observed at the rheobase, the minimum current needed to induce action potentials, was analyzed in detail. Action potential threshold was defined as the potential at which the membrane potential slope exceeded 1 V/s. Action potential latency was the time from start of the current injection to threshold. Rate of rise was the maximum voltage derivative from threshold to peak. Full width of the action potential at half maximum (FWHM) between threshold and peak, and afterhyperpolarization (AHP) time and amplitude relative to threshold were also calculated. The membrane response to hyperpolarizing current injection was quantified as the difference between the average membrane potential over 1 ms around the peak sag potential and the average membrane potential over 1 ms at the peak steady state potential. The membrane response of GnRH neurons following the termination of the hyperpolarizing current injection was quantified as the difference between the average of membrane potential for 1 ms around the peak repolarization potential and the basal membrane potential before current injection.

### Statistics

**Figure 1.**
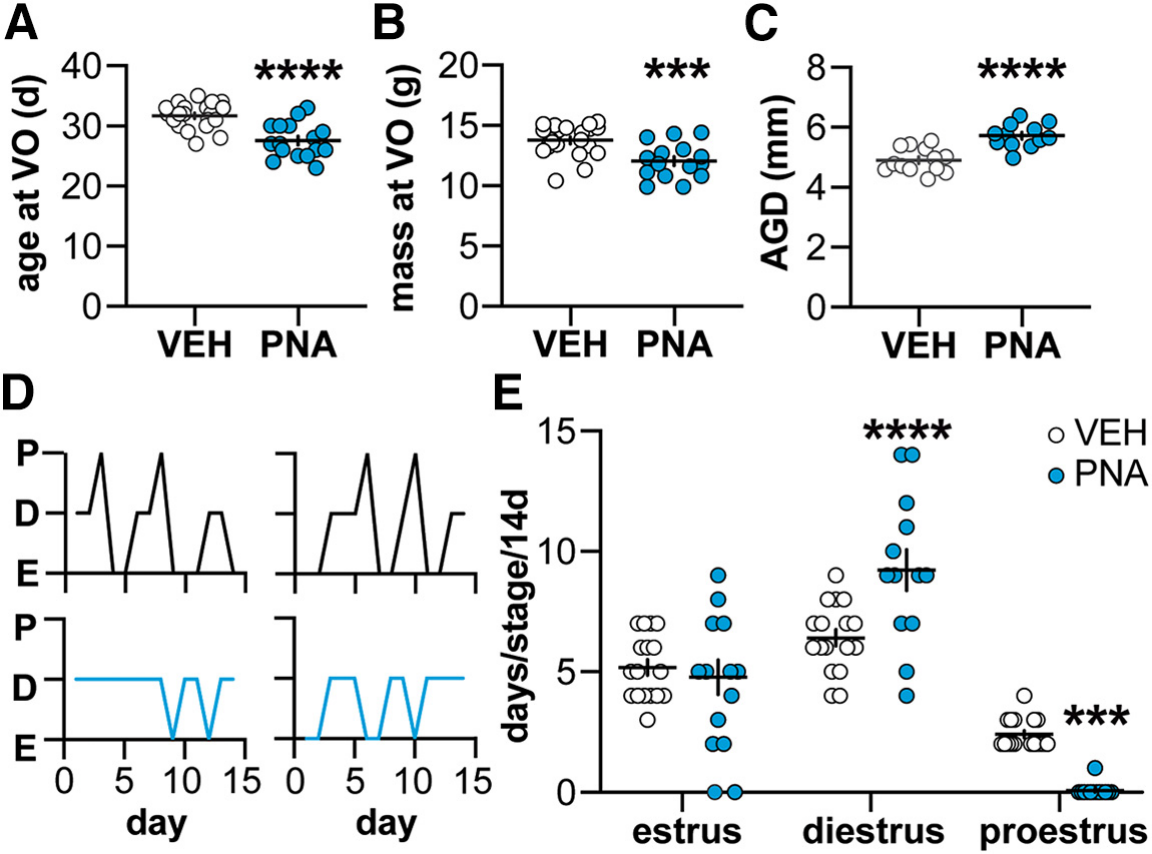
Confirmation of PNA phenotype. ***A–C***, Individual values and mean ± SEM for age of vaginal opening (VO; ***A***), body mass at VO (***B***), and adult anogenital distance (AGD, mm; ***C***). ***D***, Representative estrous cycles over 14 d. P, proestrus; D, diestrus; E, estrus. ***E***, Individual values and mean ± SEM days in each cycle stage over 14 d. Statistical parameters are in Table 1; ***p < 0.005, ****p < 0.0001.

Data are reported as mean ± SEM, with individual values shown when practical. Statistical analyses were made using Prism 9 (GraphPad Software). Data were tested for normal distribution with Shapiro–Wilk. Details of specific tests are provided in the results. Statistical tables for two-way ANOVAs report the differences in means and associated 95% confidence interval (CI) defined for age (three week-adult), treatment (control-PNA), and interaction [(adult-CON – adult-PNA) – (three-week CON – three-week PNA)]; α was set to 0.05 and *n* indicates the number of mice for Figure 1 and number of cells for electrophysiology.

**Table 1:** Descriptive statistics and statistical parameters characterizing the PNA phenotype (**Fig. 1)**

Mean ± SEM for age at VO, body mass at VO and AGD		
Property	VEH	PNA
Age at vaginal opening (d)	31.7 ± 0.5	27.6 ± 0.7
Body mass (g) at vaginal opening	13.8 ± 0.3	12.1 ± 0.4
AGD (mm)	4.9 ± 0.1	5.7 ± 0.1

Property	Unpaired, two-tailed Student’s *t* test	Mean difference (PNA-VEH)	Effect size *r*^2^
Age at vaginal opening (d)	*t*_(4.986)_, df = 34; ***p* < 0.0001**	Diff [Cl, −5.824, −2.451] −4.138 ± 0.8299	*r*^2^ = 0.4223
AGD (mm)	*t*_(5.124)_, df = 23; ***p* < 0.0001**	Diff [Cl, 0.8206, 0.1601] 0.8206 ± 0.160	*r*^2^ = 0.5331

Property	Mann–Whitney *U* test	Two-tailed *p*-value
Body mass (g) at vaginal opening	*U* = 55	***p* = 0.0006**

Mean ± SEM days per estrous cycle stage		
Cycle stage	VEH	PNA
Estrus	5.2 ± 0.3	4.5 ± 0.7
Diestrus	6.4 ± 0.3	9.2 ± 0.8
Proestrus	2.4 ± 0.2	0.1 ± 0.1

Property	χ^2^ test
Estrous cycle stage distribution	χ^2^ = 36.1672, *n *= 425, df= 2, ***p* = 0.000000014**

	Estrus	Diestrus	Proestrus
Std. residual	0.3578012	3.771665	5.7238930
Fisher’s exact test; Bonferroni adjusted	*p* = 1.0	***p* = 0.000573**	***p* < 0.00005**

Bold indicates *p* < 0.05.

## Results

### Verification of prenatal androgenization phenotype

PNA-induced differences were confirmed in the present study; for mice in which electrophysiology was done at three weeks of age and in adulthood, these aspects were verified in littermates raised to adults as the PNA phenotype is consistent among littermates. As reported (Roland and Moenter, 2011; Dulka and Moenter, 2017; Berg et al., 2018; Gibson et al., 2021), vaginal opening (VO) in the present study occurred at a younger age (unpaired, two-tailed Student’s *t* test; control *n *=* *19 mice, PNA *n *=* *16 mice) and lower body mass (two-tailed Mann–Whitney *U* test) in PNA females (Fig. 1*A*,*B*; Table 1). Anogenital distance (AGD) was increased in adult PNA mice (unpaired, two-tailed Student’s *t* test; Fig. 1*C*; Table 1; control *n *=* *15 mice, PNA *n *=* *17 mice) and estrous cycles were disrupted. Specifically, PNA females spent fewer days in proestrus and more days in diestrus (χ^2^, Fig. 1*D*,*E*; Table 1; control *n *=* *17 mice, PNA *n *=* *17 mice).

### Recording quality parameters and passive properties of GnRH neurons

**Figure 2.**
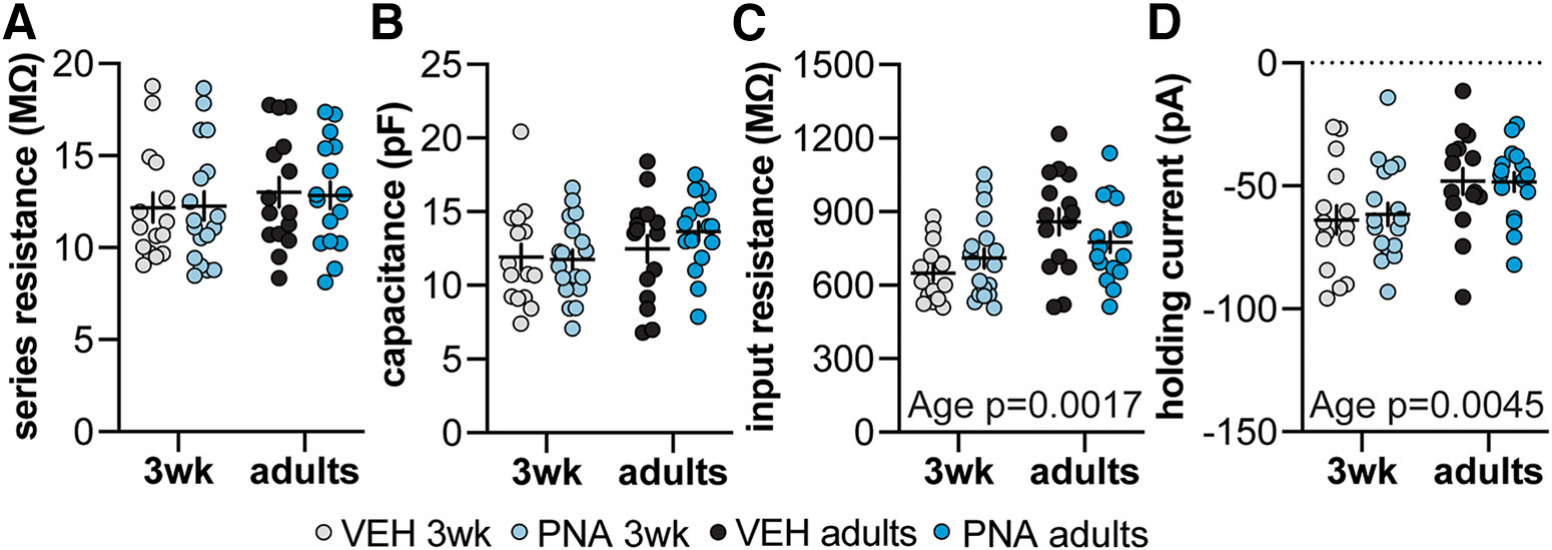
Recording quality parameters. ***A–D***, Individual values and mean ± SEM for compensated series resistance (***A***), capacitance (***B***), input resistance (***C***), holding current (***D***). Statistical parameters are in Table 2.

Passive properties and series resistance were used to assess recording quality. There were no differences among these parameters between depolarizing and hyperpolarizing current injection protocols, and they were combined for quality assessment (two-way ANOVA; three-week control *n *=* *15 cells, three-week PNA *n *=* *18 cells, adult control *n *=* *15 cells, adult PNA *n *=* *17). There were no differences in compensated series resistance or capacitance among groups (Fig. 2*A*,*B*; Table 2). Input resistance was greater and holding current was more hyperpolarized in cells from adult than three-week-old mice (Fig. 2*C*,*D*; Table 2).

**Table 2:** Descriptive statistics and statistical parameters from two-way ANOVA for recording quality parameters and passive properties (**Fig. 2)**

Descriptive statistics (mean ± SEM)				
Property	3-week controls	3-week PNA	Adult controls	Adult PNA
Series resistance (MΩ)	12.2 ± 0.8	12.259 ± 0.8	13.0 ± 0.8	12.841 ± 0.729
Capacitance (pF)	11.9 ± 0.9	11.8 ± 0.6	12.5 ± 0.9	13.654 ± 0.651
Input resistance (MΩ)	648.6 ± 30.2	710.3 ± 39.3	858.6 ± 53.4	775.946 ± 41.81
Holding current (pA)	−63.9 ± 5.8	−61.6 ± 4.6	−48.2 ± 5.3	−48.456 ± 3.820

Two-way ANOVA					
Property	Age	Treatment		Interaction	
Series resistance (MΩ)	Diff, −0.7058 [Cl, −2.250, 0.8387] *F*_(1,60)_ = 0.8356; *p* = 0.3643	Diff, 0.04112 [Cl, −1.503, 1.586] *F*_(1,60)_ = 0.002837; *p* = 0.9577		Diff, −0.2489 [Cl, −2.840, 3.338] *F*_(1,60)_ = 0.02597; *p* = 0.8725	
Capacitance (pF)	Diff, −1.220 [Cl, −2.750, 0.3089] *F*_(1,60)_ = 2.548; *p* = 0.3906	Diff, −0.5158, [Cl, −2.045, 1.014] *F*_(1,60)_ = 0.4551; *p* = 0.5025		Diff, 1.322 [Cl, −4.381, 1.736] *F*_(1,60)_ = 0.7478; *p* = 0.5025	
Input resistance (MΩ)	Diff, −137.8 [Cl, −221.8, −53.87] *F*_(1,60)_ = 10.78; ***p* = 0.0017**	Diff, 10.43 [Cl, −73.53, 94.39] *F*_(1,60)_ = 0.06174, *p* = 0.8046		Diff, −144.4 [Cl, −312.3, 23.56] *F*_(1,60)_ = 2.957; *p* = 0.0907	
	Bonferroni	3-week VEH vs 3-week PNA	3-week VEH vs adult VEH	3-week PNA vs adult PNA	Adult VEH vs adult PNA
		*p* > 0.9999	***p* = 0.0065**	*p* ≥ 0.9999	*p* > 0.9999
Holding current (pA)	Diff, −14.42 [Cl, −24.17, −4.658] *F*_(1,60)_ = 8.732; ***p* = 0.0045**	Diff, −0.9966 [Cl, −10.75, 8.761] *F*_(1,60)_ = 0.04174; *p* = 0.8388		Diff, −2.608 [Cl, −22.12, 16.91] *F*_(1,60)_ = 0.07146; *p* = 0.7901	
	Bonferroni	3-week VEH vs 3-week PNA	3-week VEH vs adult VEH	3-week PNA vs adult PNA	Adult VEH vs adult PNA
		*p* > 0.9999	*p* = 0.1846	*p* = 0.3271	*p* > 0.9999

Bold indicates *p* < 0.05.

### GnRH neurons from adult females are more excitable than those from three-week-old females

**Figure 3.**
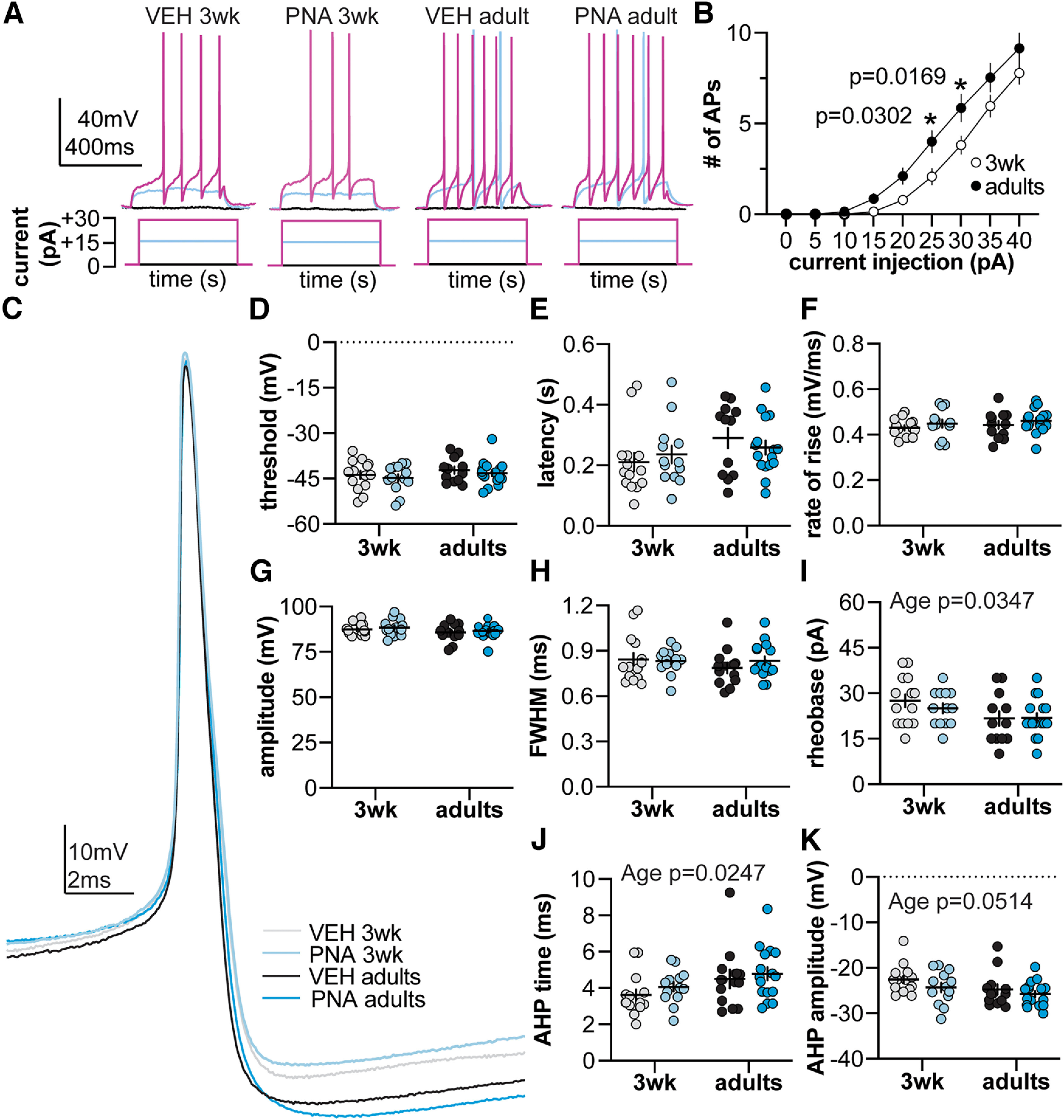
GnRH neuron excitability is increased, and action potential properties altered, in adult versus three-week-old mice. ***A***, Representative membrane voltage responses (top) to depolarizing current injections (bottom); only three current steps are shown for clarity. ***B***, Mean ± SEM # of action potentials (APs) fired as a function of current injection in age-combined groups. ***C***, representative traces of the rheobase AP for each experimental group. ***D–K***, Individual values and mean ± SEM for AP threshold (***D***), latency (***E***), rate of rise (***F***), AP amplitude (***G***), full width at half-maximum (FWHM; ***H***), rheobase (***I***), afterhyperpolarization potential (AHP) time (***J***), and AHP amplitude (***K***). Statistical parameters are in Tables 3 and 4.

Whole-cell current-clamp recordings were used to assess excitability measured as the number of action potentials generated as a function of current injection. Figure 3*A* shows representative membrane potential traces (top) in response to current injection (bottom) for each group; only three steps are shown for clarity. GnRH neurons from adult mice generated more action potentials in response to depolarizing current than those from three-week-old females, but PNA treatment had no effect on excitability (three-way, repeated-measures ANOVA, Fig. 3*B*; Table 3; three-week control *n *=* *14 cells, three-week PNA *n *=* *12 cells, adult control *n *=* *12 cells, adult PNA *n *=* *17 cells). Based on results of the three-way ANOVA, data were consolidated by age or treatment for comparison and reanalyzed by two-way, repeated-measures ANOVA, which found a difference for age and thus was followed by Bonferroni *post hoc* (Fig. 3*B*; Table 3). Age, but not treatment, consolidated data show that GnRH neurons from adult mice fire more action potentials in response to depolarizing current than those from three-week-old females.

**Table 3:** Descriptive statistics and statistical parameters from three-way repeated-measures ANOVA for GnRH neuron excitability (**Fig. 3)**

Descriptive statistics number of APs (mean ± SEM)				
Current (pA)	3-week controls	3-week PNA	Adult controls	Adult PNA
0	0.0 ± 0.0	0.0 ± 0.0	0.0 ± 0.0	0.0 ± 0.0
5	0.0 ± 0.0	0.0 ± 0.0	0.0 ± 0.0	0.0 ± 0.0
10	0.0 ± 0.0	0.0 ± 0.0	0.2 ± 0.12	0.1 ± 013
15	0.1 ± 0.14	0.2 ± 0.15	1.1 ± 0.50	0.7 ± 0.30
20	0.8 ± 0.35	0.8 ± 0.40	2.3 ± 0.75	2.0 ± 0.56
25	2.3 ± 0.70	1.8 ± 0.56	4.3 ± 1.09	3.8 ± 0.71
30	3.6 ± 0.84	4.0 ± 0.57	6.2 ± 1.36	5.6 ± 0.88
35	5.9 ± 1.10	6.1 ± 0.63	8.0 ± 1.45	7.2 ± 0.90
40	7.7 ± 1.00	7.8 ± 0.68	9.7 ± 1.51	8.8 ± 0.94

Three-way repeated-measures ANOVA		Two-way (age)	Two-way (treatment)
Current (pA)	*F*_(8,408)_ = 176.9; ***p* < 0.0001**	*F*_(1.341,71.06)_ = 183.1; ***p* < 0.0001**	*F*_(1.348,71.46)_ = 173.9; ***p* < 0.0001**
Age	*F*_(1,51)_ = 4.515; ***p* = 0.0385**	*F*_(1,53)_ = 4.478; ***p* = 0.0391**	
Treatment	*F*_(1,51)_ = 0.1273; *p* = 0.7227		*F*_(1,53)_ = 0.02802; *p* = 0.8677
Current × age	*F*_(8,408)_ = 2.977; ***p* = 0.0030**	*F*_(8,424)_ = 2.974; ***p* = 0.0030**	
Current × treatment	*F*_(8,408)_ = 0.120; *p* = 0.9984		*F*_(8,424)_ = 0.06764; *p* = 0.9998
Age × treatment	*F*_(1,51)_ = 0.1738; *p* = 0.1738		
Current × age × treatment	*F*_(8,408)_ = 0.228; *p* = 0.9857		

Bonferroni of age-consolidated data	Three weeks (CON and PNA) vs adult (CON and PNA)
0, 5, 10, 15 pA	*p* > 0.9999
20 pA	*p* = 0.3820
25 pA	*p* = 0.0302
30 pA	*p* = 0.0169
35 pA	*p* = 0.1481
40 pA	*p* = 0.3349

Bold indicates *p* < 0.05.

**Table 4:** Descriptive statistics for action potential properties (**Fig. 3)**

Descriptive statistics (mean ± SEM)				
Property	3-week controls	3-week PNA	Adult controls	Adult PNA
Threshold (mV)	−43.8 ± 1.31	−44.8 ± 1.24	−42.2 ± 1.17	−43.2 ± 0.95
Latency (s)	0.2 ± 0.11	0.2 ± 0.10	0.3 ± 0.12	0.3 ± 0.10
Amplitude (mV)	87.5 ± 0.84	88.4 ± 1.28	85.7 ± 1.46	86.7 ± 1.05
FWHM (ms)	0.8 ± 0.04	0.8 ± 0.02	0.8 ± 0.04	0.8 ± 0.03
Rate of rise (mV/ms)	0.4 ± 0.01	0.4 ± 0.02	0.4 ± 0.02	0.5 ± 0.01
Rheobase (mV)	27.5 ± 2.15	25.0 ± 1.60	21.7 ± 2.41	21.9 ± 1.57
AHP amplitude (mV)	−22.6 ± 0.85	−24.3 ± 1.02	−24.8 ± 1.15	−25.8 ± 0.68
AHP time (ms)	3.6 ± 0.31	4.1 ± 0.26	4.5 ± 0.52	4.8 ± 0.36

### Developmental stage alters GnRH neuron action potential properties

To generate hypotheses about specific ionic current changes that may underlie the above observations, we examined properties of the first spike generated at rheobase (Tables 4, 5). Representative traces aligned by AP threshold are shown in Figure 3*C* for each group. There were no differences in AP threshold, latency, rate of rise, amplitude, or FWHM among groups (two-way ANOVA; Fig. 3*D–H*). The rheobase was lower in cells from adult mice, consistent with the increased excitability and input resistance (Fig. 3*I*). Despite the similarity of the spike phase of the AP, the AHP differed among groups. Specifically, the AHP peaked later in adult compared with three-week-old females regardless of PNA treatment (Fig. 3*J*), and the amplitude approached the level set for significance for being larger in adults (Fig. 3*K*). Together, these observations suggest that changes in the action potential properties of GnRH neurons are primarily driven by development.

**Table 5:** Two-way ANOVA parameters for action potential properties (**Fig. 3)**

Property	Age	Treatment		Interaction	
Threshold (mV)	Diff, 1.170 [Cl, −3.918, 0.7768] *F*_(1,52)_ = 1.803; *p* = 0.1852	Diff, 0.9859 [Cl, −1.361, 3.333] *F*_(1,52)_ = 0.7103; *p* = 0.4032		Diff, −0.01028 [Cl, −4.705, 4.684] *F*_(1,52)_ = 1.932e-005; *p* = 0.9965	
Latency (s)	Diff, −0.05101 [Cl, −0.1089, 0.006901] *F*_(1,51)_ = 0.3.127; *p* = 0.0.0830	Diff, 0.003030 [Cl, −0.05489, 0.06095] *F*_(1,51)_ = 0.0.01103; *p* = 0.9168		Diff, −0.05693 [Cl, −0.1728, 0.05890] *F*_(1,51)_ = 0.9735; *p* = 0.3285	
Amplitude (mV)	Diff, 1.731 [Cl, −0.5921, 4.055] *F*_(1,51)_ = 2.238; *p* = 0.1408	Diff, −0.9231 [Cl, −3.247, 1.400] *F*_(1,51)_ = 0.0.6361; *p* = 0.4288		Diff, 0.002851 [Cl, −4.650,4.644] *F*_(1,51)_ = 1.517e006; *p* = 0.9990	
FWHM (ms)	Diff, 0.02731 [Cl, −0.03989, 0.09454] *F*_(1,51)_ = 0.6660; *p* = 0.4183	Diff, −0.01879 [Cl, −0.8601, 0.04843] *F*_(1,51)_ = 0.3149; *p* = 0.5771		Diff, 0.05826 [Cl, −0.07618, 0.1927] *F*_(1,51)_ = 0.7568; *p* = 0.3884	
Rate of rise (mV/ms)	Diff, −0.01261 [Cl, −0.04178, 0.01657] *F*_(1,51)_ = 0.7524; *p* = 0.3898	Diff, 0.01453 [Cl, −0.04740, 0.01095] *F*_(1,51)_ = 0.2155; *p* = 0.2155		Diff, −0.0002991 [Cl, −0.05865, 0.05805] *F*_(1,51)_ = 0.0001059; *p* = 0.9918	
Rheobase (pA)	Diff, 4.479 [Cl, 0.5934, 8.365] *F*_(1,51)_ = 5.355; ***p* = 0.0247**	Diff, 1.146 [Cl, −2.740, 5.032] *F*_(1,51)_ = 0.3505; *p* = 0.5565		Diff, 2.708 [Cl, −5.063, 10.48] *F*_(1,51)_ = 0.4895; *p* = 0.4873	
	Bonferroni	3-week VEH vs 3-week PNA	3-week VEH vs adult VEH	3-week PNA vs adult PNA	Adult VEH vs adult PNA
		*p* > 0.9999	*p* = 0.2568	*p* > 0.9999	*p* > 0.9999
AHP amplitude (mV)	Diff, 1.827 [Cl, −0.01161, 3.666] *F*_(1,51)_ = 3.980**;** *p* = 0.0514	Diff, 1.369 [Cl, −0.4701, 3.208] *F*_(1,51)_ = 2.233; *p* = 0.1412		Diff, 0.7832 [Cl, −2.895, 4.461] *F*_(1,51)_ = 0.1827; *p* = 0.6708	
	Bonferroni	3-week VEH vs 3-week PNA	3-week VEH vs adult VEH	3-week PNA vs adult PNA	Adult VEH vs adult PNA
		*p* > 0.9999	*p* = 0.6064	*p* > 0.9999	*p* > 0.9999
AHP time (ms)	Diff, −0.7990 [Cl, −1.538, −0.05992] *F*_(1,51)_ = 4.710; ***p* = 0.0347**	Diff, −0.3644 [Cl, −1.103, 0.3747] *F*_(1,51)_ = 0.9797; *p* = 0.3270		Diff, −0.1663 [Cl, −1.644, 1.312] *F*_(1,51)_ = 0.05100; *p* = 0.8222	
	Bonferroni	3-week VEH vs 3-week PNA	3-week VEH vs adult VEH	3-week PNA vs adult PNA	Adult VEH vs adult PNA
		*p* > 0.9999	*p* = 0.6283	*p* = 0.9836	*p* > 0.9999

Bold indicates *p* < 0.05.

### There are no differences in the response of GnRH neurons to hyperpolarizing current during development or with PNA treatment

**Figure 4.**
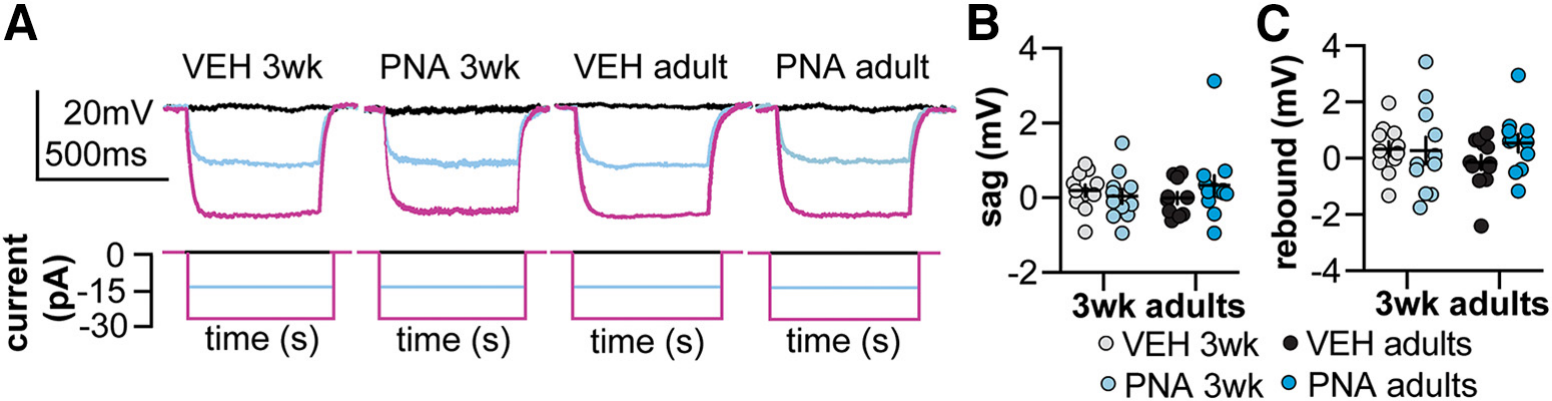
Neither development nor PNA treatment alter the response of GnRH neurons to hyperpolarizing current. ***A***, Representative membrane voltage (top) responses to hyperpolarizing current injections (bottom); only three steps are shown for clarity. Individual values ± SEM for sag (***B***), and GnRH neuron rebound following the hyperpolarizing current relative to baseline membrane potential (***C***). Statistical parameters shown in Table 5.

Representative membrane voltage traces (top) in response to the hyperpolarizing current are shown in Figure 4*A*; only three current steps are shown for clarity. No cells exhibited rebound spikes in response to termination of hyperpolarizing current injection. To assess membrane sag typically associated with activation of hyperpolarization-activated current (Ih), comparisons were made among cells that were hyperpolarized to a membrane potential between −90 and −95 mV. Negative current between −10 to −45 pA was needed to hyperpolarize GnRH neurons to this membrane potential range and less hyperpolarizing current was required to achieve this membrane potential in adulthood compared with three-week-old females regardless of PNA treatment (Table 6; three-week control *n *=* *11 cells, three-week PNA *n *= 10 cells, adult control *n *=* *11 cells, adult PNA *n *=* *13 cells). The membrane potential difference between peak of the sag and steady-state (sag) was both minimal and similar among experimental groups (Fig. 4*B*; Table 6). There was also no difference in GnRH neuron rebound depolarization after termination of the current injection (Fig. 4*C*; Table 6). These data suggest neither development nor PNA treatment alter the response of GnRH neurons to hyperpolarizing input within the range tested.

**Table 6:** Mean ± SEM and two-way ANOVA parameters for GnRH neurons hyperpolarized between −90 and −95 mV (**Fig. 4)**

Current (pA) needed to hyperpolarize the cell to −90 to −95 mV			
VEH 3 weeks	PNA 3 weeks	VEH adult	PNA adult
−37.3 ± 1.95	−35.0 ± 1.78	−31.0 ± 2.67	−31.5 ± 1.73

Steady-state to sag-peak membrane potential difference			
VEH 3 weeks	PNA 3 weeks	VEH adult	PNA adult
0.2 ± 0.12	0.04 ± 0.20	−0.01 ± 0.15	0.3 ± 0.26

Repolarization to basal membrane potential difference			
VEH 3 weeks	PNA 3 weeks	VEH adult	PNA adult
0.3 ± 0.26	0.3 ± 0.48	−0.1 ± 0.26	0.5 ± 0.32

Two-way ANOVA					
Property	Age	Treatment		Interaction	
Current (pA) needed to hyperpolarizethe cell to −90 to −95 mV	Diff, −4.867 [Cl, −8.960, −0.7745] *F*_(1,41)_ = 5.768; ***p* = 0.0209**	Diff, −0.8671 [Cl, −4.960, 3.225] *F*_(1,41)_ = 0.1831; *p* = 0.6710		Diff, −2.811 [Cl, −11.0, 21.915.374 *F*_(1,41)_ = 0.4811; *p* = 0.4918	
	Bonferroni	3-week VEH vs 3-week PNA	3-week VEH vs adult VEH	3-week PNA vs adult PNA	Adult VEH vs adult PNA
		*p* > 0.9999	*p* = 0.2398	*p* > 0.9999	*p* > 0.9999
Steady-state to sag-peak potential difference	Diff, −0.04333 [Cl, −0.4615, 0.3748] *F*_(1,41)_ = 0.04380; *p* = 0.8353	Diff, −0.09561 [Cl, −0.5138, 0.00928503226] *F*_(1,41)_ = 0.2132; *p* = 0.6467		Diff, 0.4902 [Cl, −0.3461, 1.327] *F*_(1,41)_ = 1.401; *p* = 0.2433	
Repolarization to basal potential difference	Diff, 0.09892 [Cl, −0.5909, 0.7887] *F*_(1,41)_ = 0.08386; *p* = 0.7736	Diff, −0.3164 [Cl, −1.006, 0.3735] *F*_(1,41)_ = 0.8579; *p* = 0.3597		Diff, 0.7403 [Cl, −0.6393, 2.120] *F*_(1,41)_ = 1.174; *p* = 0.2848	

Bold indicates *p* < 0.05.

## Discussion

Reproduction is centrally controlled by the release pattern of GnRH. Disruptions in GnRH release are postulated to occur in hyperandrogenemic women with PCOS based on the observed increased frequency of pulsatile LH secretion. We used a mouse model that recapitulates the neuroendocrine aspects of PCOS to test the hypotheses that intrinsic properties of GnRH neurons change during typical development, and that these developmental changes are altered by PNA treatment (Roland and Moenter, 2011; Moore et al., 2015; Dulka and Moenter, 2017; Berg et al., 2018). Data supported the first hypothesis but rejected the second as the intrinsic properties of GnRH neurons tested change with age but are not affected by PNA treatment. Specifically, GnRH neurons from adult mice are more excitable and have altered action potential properties compared with those from three-week-old mice.

During typical development, the spontaneous action potential firing rate of GnRH neurons is dynamic, with mean GnRH neuron firing rate peaking at three weeks of age before decreasing to lower levels in adulthood (Roland and Moenter, 2011; Dulka and Moenter, 2017). In contrast, the firing rate in GnRH neurons from PNA mice is steady throughout development. As a result, firing rate of GnRH neurons from PNA mice is lower than controls at three weeks of age, but increased in adulthood (Roland and Moenter, 2011; Dulka and Moenter, 2017). These observations indicate that both development and PNA treatment play a role in shaping the activity of these neurons. This overall firing rate is shaped by a combination of the intrinsic properties of and synaptic inputs to GnRH neurons, as well as interactions with non-neuronal cells like glia (Prevot and Sharif, 2022). The present work suggests that the intrinsic properties of these neurons are developmentally regulated but not affected by PNA treatment. This finding is consistent with work in which transcriptome profiling of GnRH neurons from the same groups revealed more developmental changes than changes that were induced by PNA treatment (Burger et al., 2020). Whereas GnRH neuron membrane response to depolarizing current changed between the ages studied, there were no differences among groups in membrane response to hyperpolarizing current or in rebound firing.

To begin to formulate hypotheses about the changes that might underlie the observed postpubertal increase in GnRH neuron excitability, properties of the first action potential were measured. In adulthood, GnRH neurons also have increased input resistance, which likely is a strong contributor to two other changes observed with development, specifically increased excitability and reduced amount of current needed to induce firing (rheobase). Cells from adults also had a larger and delayed peak of the afterhyperpolarization potential regardless of PNA treatment. Changes to voltage-gated channels likely underlie these developmental differences. Potassium currents are major regulators of neuronal excitability and also sculpt the afterhyperpolarization potential. Blocking 4-aminopyridine-sensitive potassium channels in dorsal root ganglion neurons decreases the latency to fire in response to current injection and increases firing frequency (Vydyanathan et al., 2005). In motor neurons derived from patients with amyotrophic lateral sclerosis, partial pharmacological block of voltage-gated potassium currents reduces neuronal hypoexcitability and restores typical firing patterns (Naujock et al., 2016), and in cerebellar granule cells, a transient A-type potassium current increases during development concomitant with an increase in rheobase and spike latency (Shibata et al., 2000).

Similar effects of voltage-gated potassium conductances on excitability have been observed in hypothalamic neurons regulating reproduction. Estradiol-dependent increases in the membrane response to GABA and AMPA conductances applied with dynamic clamp in arcuate kisspeptin neurons were attributable to reduced A-type potassium currents (DeFazio et al., 2019). Consistent with this, increasing the A-type potassium current in these neurons with dynamic clamp made firing irregular and increased the interval between action potentials (Mendonça et al., 2018). In GnRH neurons from adults, the A-type potassium current is reduced in a model of estradiol positive feedback (ovariectomized plus estradiol) compared with cells from ovariectomized mice (DeFazio and Moenter, 2002). This was associated with a reduced latency to action potential firing (DeFazio and Moenter, 2002) and may contribute to the increase in spontaneous firing rate observed during positive feedback (Christian et al., 2005; Silveira et al., 2017). These observations, along with the delayed AHP, increased AHP amplitude and differences in rheobase and excitability observed in the current study suggest that there may be developmental changes to potassium currents in GnRH neurons.

In addition to the voltage-gated potassium currents mentioned above, voltage-gated sodium currents are critical for the initiation and propagation of action potentials, and also regulate neuronal excitability (Armstrong and Hille, 1998; Hille, 2001). In animal models of sensory neuropathies, mutations impairing fast inactivation of voltage-gated sodium channels increase excitability, hyperpolarize action potential threshold, and increase action potential FWHM (Xiao et al., 2019). In the present study, the lack of differences in these parameters, despite the differences in excitability, suggests that any changes in voltage-gated sodium channels in GnRH neurons from the groups studied are likely subtle. Voltage-gated calcium channels can also regulate neuronal excitability (Simons, 1988; Simms and Zamponi, 2014). Inhibition of T-type voltage-gated calcium channels in a subpopulation of medial habenula neurons reduces rebound burst firing and excitability (Vickstrom et al., 2020). Activation of voltage-gated calcium channel families can subsequently activate calcium-dependent potassium channels (Armstrong and Matteson, 1986; Storm, 1987), which can contribute to the shape and timing of the AHP and thus sculpt the firing activity of neurons (Sah and Faber, 2002). Calcium-dependent potassium currents have also been detected in GnRH neurons from mice and guinea pigs; these can be modulated by estradiol feedback in mice, shape the AHP and contribute to both firing patterns and subthreshold oscillations in these cells (Bosch et al., 2002; Liu and Herbison, 2008; Chu et al., 2012). Direct studies of specific currents will be needed to address these questions as changes to one or more voltage-gated currents cannot be ruled out by the present study, nor can PNA-induced changes despite the lack of effect of this treatment on overall excitability as quantified.

Development and PNA treatment could have independent and interacting effects on GnRH neuron activity and thus downstream reproductive function. The developmental increases in GnRH neuron excitability observed in the present study may help drive the pubertal reawakening of the reproductive neuroendocrine system. This may indicate intrinsic properties favoring firing become more important for effective neuroendocrine output in adults, perhaps because of concomitant changes in synaptic inputs. In PNA mice, the differences in spontaneous GnRH neuron firing (Dulka and Moenter, 2017) combined with the lack of PNA effects on stimulus-induced firing observed in this study focusing on overall intrinsic properties of these cells might indicate that it is primarily mechanisms upstream of GnRH neurons that are engaged by PNA treatment to increase spontaneous firing rate of these cells in adults. In this regard, PNA treatment increases GABAergic neurotransmission to GnRH neurons at three weeks of age and in adulthood and increases appositions on GnRH neurons by GABAergic afferents originating in part from the arcuate hypothalamus (Moore et al., 2015). Increased activation of GABAergic afferents to GnRH neurons increases LH levels and LH pulse frequency (Silva et al., 2019). While LH is not a direct measure of GnRH neuron activity, pulse frequency is a good bioassay under most circumstances (Moenter, 2015). Together, these observations suggest increased GABAergic input to GnRH neurons correlates with increased GnRH neuron activity.

Other intrinsic mechanisms could contribute to altered response to GABA. First, it is possible that PNA treatment alters chloride cotransporter function. At the soma, there were no differences in the reversal potential of GABA_A_-receptor-mediated current between control and PNA females at three weeks of age (Berg et al., 2018). It is not known, however, whether differences exist in the chloride homeostasis along GnRH neuron projections; if there is a lower intracellular chloride along the processes, for example, this could blunt the influence of more distal inputs. Second, PNA treatment may alter the number, type or subcellular location of GABA_A_-receptors on GnRH neurons. One study in adults showed that PNA increases the amplitude of GABAergic postsynaptic currents and alters their kinetics in adulthood (Sullivan and Moenter, 2004), while another study did not show these alterations through development although a trend toward higher amplitude PSCs in adults was observed (Berg et al., 2018). It is important to bear in mind that these measures are made in the soma and that changes that occur in distal cell compartments may not be detected.

It is also possible that other afferents, including glutamate and neuropeptides, are altered by PNA treatment. In this regard, neurons in the hypothalamic arcuate nucleus called KNDy neurons use glutamate, kisspeptin, neurokinin B, and dynorphin to communicate. KNDy neurons are postulated to serve as a GnRH-pulse generator (Han et al., 2015). Of note, the frequency of LH pulses in female rodents and in both male and female sheep is increased by PNA treatment (Veiga-Lopez et al., 2008; Recabarren et al., 2012; Yan et al., 2014; Silva et al., 2019). KNDy neurons express receptors for gonadal steroids (Ruka et al., 2016; Silva et al., 2019), and thus serve as an important site for steroidal feedback modulation of GnRH neuron activity and a possible site of androgen action for the PNA phenotype (Pielecka-Fortuna et al., 2011; Yeo et al., 2014; Adams et al., 2018; Nagae et al., 2021). KNDy neurons have been studied in PNA mice, and while both GABAergic and glutamatergic appositions to these cells are reduced in PNA mice (Moore et al., 2021), neither the spontaneous firing rate nor the burst firing patterns of KNDy neurons change with development or with PNA treatment (Gibson et al., 2021). KNDy-mediated changes in input to GnRH neurons could be generated by alterations in neuromodulator expression (Goodman et al., 2013; Ahn et al., 2015). How other afferents to GnRH neurons change throughout development and/or with PNA treatment and whether they shape GnRH neuron firing remains to be studied.

Here, we demonstrate that changes to overall GnRH neuron excitability and action potential properties are primarily driven by age, and not altered by prenatal androgen exposure. Future work will focus on how specific ion channels are altered and how these changes shape the response of GnRH neurons to the input they receive. These findings contribute to the overall knowledge of GnRH neurons, how their intrinsic properties are shaped during development and with PNA treatment and generate testable hypotheses as to the cause of these developmental changes.

## Synthesis

Reviewing Editor: Julie Bakker, University of Liege Decisions are customarily a result of the Reviewing Editor and the peer reviewers coming together and discussing their recommendations until a consensus is reached. When revisions are invited, a fact-based synthesis statement explaining their decision and outlining what is needed to prepare a revision will be listed below. The following reviewer(s) agreed to reveal their identity: Richard Piet, Anne-Simone Parent.

synthesis: two reviewers have reviewed the ms and both acknowledge the quality of the studies. They have however some comments/suggestions to improve the ms, please see below:

Reviewer 1:

In this short manuscript, the authors report on the excitability of mouse gonadotropin-releasing hormone (GnRH) neurons in brain slices from juvenile and adult females treated prenatally with androgen hormones (PNA; an animal model of polycystic ovary syndrome PCOS) or vehicle (control). The authors hypothesized that GnRH neuron excitability and action potential properties would vary across the pubertal transition and that this would be altered by PNA. Results indicate that GnRH neurons are more excitable and display subtle changes in action potential afterhyperpolarization potentials (AHP) in brain slices from adults compared with slices from juvenile mice. This, however, is not altered by PNA.

The manuscript is relatively straightforward and is easy to read. The work is based on a logical hypothesis and will add to the current knowledge of the effects of PNA on GnRH neuron activity in models of PCOS. The methodology and experimental designs are sound and clearly presented. The illustrations are overall very clear with high quality example traces.

My comments and criticisms are editorial and relatively minor, and are listed below.

- My main criticism relates to how results are presented in the text. Whereas specifics about results of statistical tests are provided in the tables, the authors do not give the readers any numerical values describing the actual data (mean and error, data range) - although data points and means are displayed in the figures, detailing descriptive statistics in the text would be beneficial (and save readers from using a ruler!).

- The title of the manuscript and of figure 3 may be overstated when it comes to alterations of action potential properties: unless this reviewer is missing something, the only statistically significant change is a relatively subtle change (hard to judge without the mean values) in the timing of the AHP peak, whereas other properties measured were unchanged. Please revise.

- This reviewer missed a discussion of the potential physiological implications of the authors’ results for the reactivation of GnRH secretion during the pubertal transition.

- Likewise, it may be interesting to discuss the observations that excitability increases in GnRH across the pubertal transition, when spontaneous firing decreases.

Other comments:

- The paragraph between lines 59-73 reads “discussion-y” and could be shortened.

- In the introduction, the hypotheses should be stated earlier, perhaps around line 73. The hypotheses also do not fully match, as written, in the introduction and in the discussion (lines 221-223)

- Line 87, is it important to specify “Eastern Standard Time”?

- Line 94, “[Author University]”?

- Methods, brain slice preparation: maybe indicate the range of time after lights on animals were killed.

- Line 106, Whole-cell current-clamp patch clamp electrophysiology?

- Line 135-136, suggest “Current steps (500ms, 5pA increments from -50 to 40 pA)...”; from 0pA to -50pA cannot be achieved in increments of 5 pA.

- Lines 143-149, please revise to explain what was measured, i.e. the presence or absence of a sag depolarization or of a rebound depolarization.

- Line 167, χ</sup>2

- Line 182, Figure 3B does not show control vs PNA - please revise

- Line 195, depolarizing phase of the AP?

- Lines 247-255, the authors discuss the role of potassium conductances in other neurons - they may also refer to work by the Herbison group on potassium currents in GnRH neurons and their impact on firing patterns.

- In the discussion paragraph starting line 283, the authors may note that increased GnRH neuron firing and increased GABAergic synaptic transmission in PNA have not been formally linked causally, to this reviewer’s knowledge.

- Table 1: mean difference values are missing for age at VO and AGD

- Table 3 is confusing, especially the results of Bonferroni tests for consolidated two-way ANOVA. Please revise

- Table 4: column Age/row Latency, please revise p value; in addition, why report the results of Bonferroni analyses if no significant differences were detected by the two-way ANOVA analysis?

- Figure 2-4 may have too many colors for data, seeing that data are presented as 3 week old vs adult - two colors (for control and PNA) may be enough.

- Figure 3C: the key for the traces is dots of different colors. These should be lines of different colors.

- Figure 3I and J: please give actual p values to match B and K and Figure 2

- Figure 4A: the current scale is wrong, please revise to show 0 at top and -30 at bottom of scale.

Reviewer 2:

The authors measured GnRH neuron excitability and action potential properties in control and postnatally androgenized female mice before puberty and in adulthood using whole-cell current clamp recording in brain slices. Through a straightforward set of experiments combined with well described statistical analysis, they showed that development but not prenatally androgenization is a driver for changes in GnRH neuron intrinsic properties. Their data suggests that developmentally-induced changes in voltage-gated ion channels in GnRH neurons could explain the changes in response to synaptic input. Indirectly, it supports mechanisms upstream of GnRH neurons to explain alterations in spontaneous firing rate after postnatal androgenization.

Specific comments:

- The abstract contains some inacuracies: “symptoms resembling PCOS are observed in girls at or near the time of pubertal onset” What do the authors mean by this affirmation?

- Regarding brain slice preparations from adult mice, the day of the estrus cycle on which the experiment was conducted should be specified.

- The authors nicely document the efficiency of their model (vaginal opening, estrus cyclicity etc) but data reagarding LH levels, LH pulsatility or testosterone levels (even as a citation of previous study) is missing. In particular, testosterone levels in prepubertal animals would be interesting to document. Moreover, such PNA model does not always lead to increased androgen levels in adults.

## Author Response

We thank the editorial staff and reviewers for their time and effort in handling and providing constructive feedback on our manuscript. We have used this feedback to improve the manuscript. Below, we provide a point-by-point response to the reviewer comments in blue.

Changes within the manuscript are in indicated in bold synthesis: two reviewers have reviewed the ms and both acknowledge the quality of the studies.

They have however some comments/suggestions to improve the ms, please see below:

Reviewer 1:

In this short manuscript, the authors report on the excitability of mouse gonadotropin-releasing hormone (GnRH) neurons in brain slices from juvenile and adult females treated prenatally with androgen hormones (PNA; an animal model of polycystic ovary syndrome PCOS) or vehicle (control). The authors hypothesized that GnRH neuron excitability and action potential properties would vary across the pubertal transition and that this would be altered by PNA.

Results indicate that GnRH neurons are more excitable and display subtle changes in action potential afterhyperpolarization potentials (AHP) in brain slices from adults compared with slices from juvenile mice. This, however, is not altered by PNA.

The manuscript is relatively straightforward and is easy to read. The work is based on a logical hypothesis and will add to the current knowledge of the effects of PNA on GnRH neuron activity in models of PCOS. The methodology and experimental designs are sound and clearly presented. The illustrations are overall very clear with high quality example traces.

My comments and criticisms are editorial and relatively minor, and are listed below.

- My main criticism relates to how results are presented in the text. Whereas specifics about results of statistical tests are provided in the tables, the authors do not give the readers any numerical values describing the actual data (mean and error, data range) - although data points and means are displayed in the figures, detailing descriptive statistics in the text would be beneficial (and save readers from using a ruler!).

We have added the mean and standard error of the mean to the tables.

- The title of the manuscript and of figure 3 may be overstated when it comes to alterations of action potential properties: unless this reviewer is missing something, the only statistically significant change is a relatively subtle change (hard to judge without the mean values) in the timing of the AHP peak, whereas other properties measured were unchanged. Please revise.

In addition to the differences in the AHP peak, GnRH neurons from adult mice fire more action potentials in response to depolarizing current compared to 3-wk-old mice, have a reduced rheobase and a small shift in AHP amplitude. All of these changes are dependent upon age but not PNA treatment. While other action potential properties remained unchanged, the differences observed suggest that there are developmental but not PNA induced changes to the excitability and action potential properties of GnRH neurons.

- This reviewer missed a discussion of the potential physiological implications of the authors’ results for the reactivation of GnRH secretion during the pubertal transition.

- Likewise, it may be interesting to discuss the observations that excitability increases in GnRH across the pubertal transition, when spontaneous firing decreases.

Thank you for these two suggestions. We’ve addressed these points in the discussion by adding

the following statements:

"Development and PNA treatment could have independent and interacting effects on GnRH

neuron activity and thus downstream reproductive function. The developmental increases in

GnRH neuron excitability observed in the present study may help drive the pubertal

reawakening of the reproductive neuroendocrine system. This may indicate intrinsic properties

favoring firing become more important for effective neuroendocrine output in adults, perhaps

because of concomitant changes in synaptic inputs. In PNA mice, the differences in

spontaneous GnRH neuron firing27 combined with the lack of PNA effects on stimulus-induced

firing observed in this study focusing on overall intrinsic properties of these cells might indicate

that it is primarily mechanisms upstream of GnRH neurons that are engaged by PNA treatment

to increase spontaneous firing rate of these cells in adults.” (Lines 293-302).

Other comments:

- The paragraph between lines 59-73 reads “discussion-y” and could be shortened.

We intentionally made the introduction a bit more in depth because many of our colleagues who

are interested in this animal model are not doing electrophysiologic studies and we wanted to

explain the rationale for the studies. The word count is below that of the journal limits, so we

have kept the original version.

- In the introduction, the hypotheses should be stated earlier, perhaps around line 73. The

hypotheses also do not fully match, as written, in the introduction and in the discussion (lines

221-223)

We have added a postulate earlier in the development of the story in the introduction to help

guide the reader towards the hypotheses that are ultimately proposed at the end of the

introduction.

- Line 87, is it important to specify “Eastern Standard Time”?

We specify this to let readers know that our experimental times remain the same regardless of

daylight savings time.

- Line 94, “[Author University]”?

In accordance with the double-blind reviewing process, we used the following statement

regarding animal use “All animal procedures were performed in accordance with the [Author

University] animal care committee’s regulations.” that is suggested in the manuscript submission

guidelines.” (Lines 96-98).

- Methods, brain slice preparation: maybe indicate the range of time after lights on animals were

killed.

Thanks, his information has been added (Lines 100-101)

- Line 106, Whole-cell current-clamp patch clamp electrophysiology?

We reworded this to read “Whole-cell patch-clamp recordings in current-clamp mode were

conducted to investigate the intrinsic properties of GnRH neurons of PNA and control mice

before and after puberty” (Lines 111-113).

- Line 135-136, suggest “Current steps (500ms, 5pA increments from -50 to 40 pA)...”; from 0pA

to -50pA cannot be achieved in increments of 5 pA.

Excellent catch! We have specified that increments or decrements were used depending on the

change in direction of the current steps.

"Current steps (500ms, 5pA increments from 0pA to +40pA, 5pA decrements from 0pA to -

50pA) were delivered to test the membrane potential response.” (Lines 140-142).

- Lines 143-149, please revise to explain what was measured, i.e. the presence or absence of a

sag depolarization or of a rebound depolarization.

We have clarified this as follows, “The membrane response to hyperpolarizing current injection

was quantified as the difference between the average membrane potential over 1ms around the

peak sag potential and the average membrane potential over 1ms at the peak steady state

potential. The membrane response of GnRH neurons following the termination of the

hyperpolarizing current injection was quantified as the difference between the average of

membrane potential for 1ms around the peak repolarization potential and the basal membrane

potential before current injection.”

- Line 167, χ2

Thank you for pointing out this detail. It has been fixed. (Line 172)

- Line 182, Figure 3B does not show control vs PNA - please revise

The initial three-way ANOVA run, which is detailed in Table 3, showed an age and an age x

current effect but no effect of PNA treatment. Because of this, we performed a standard

consolidation of the groups by age for a two-way ANOVA, these are the data shown in Figure 3.

All tests and parameters are in Table 3.

We have revised the text on this to clarify as follows: “Based on results of the three-way

ANOVA, data were consolidated by age or treatment for comparison and reanalyzed by two way, repeated-measures ANOVA, which found a difference for age thus was followed by

Bonferroni post hoc (Figure 3B, Table 3). Age, but not treatment, consolidated data show that

GnRH neurons from adult mice fire more action potentials in response to depolarizing current

than those from 3-wk females”. (Lines 189-191)

- Line 195, depolarizing phase of the AP?

We used the phrase “spike phase” because the traces are near overlapping for the rapid

depolarizing and most of the rapid depolarization, i.e., more than just during depolarization

- Lines 247-255, the authors discuss the role of potassium conductances in other neurons - they

may also refer to work by the Herbison group on potassium currents in GnRH neurons and their

impact on firing patterns.

Thank you for this suggestion. We have added the following statement to the discussion:

"Calcium-dependent potassium currents have also been detected in GnRH neurons from mice

and guinea pigs; these can be modulated by estradiol feedback in mice, shape the AHP and

contribute to both firing patterns and subthreshold oscillations in these cells56-58.” (Lines 286-

289)

- In the discussion paragraph starting line 283, the authors may note that increased GnRH

neuron firing and increased GABAergic synaptic transmission in PNA have not been formally

linked causally, to this reviewer’s knowledge.

The reviewer is correct that a direct link has not been made and we have clarified this in the

discussion by adding the following:

"Increased activation of GABAergic afferents to GnRH neurons increase LH concentration levels

and LH pulse frequency58. While LH is not a direct measure of GnRH neuron activity, it is a

good bioassay under most circumstances59. Together, these observations suggest increased GABAergic input to GnRH neurons correlates with increased GnRH neuron activity.” (Lines 305-

309)

- Table 1: mean difference values are missing for age at VO and AGD

We have added the mean differences values {plus minus} SEM for age at VO and AGD. (Table 1)

- Table 3 is confusing, especially the results of Bonferroni tests for consolidated two-way

ANOVA. Please revise

We have revised Table 3 and have indicated that the Bonferroni test reflects the age consolidated two-way ANOVA data. See table below.

- Table 4: column Age/row Latency, please revise p value; in addition, why report the results of

Bonferroni analyses if no significant differences were detected by the two-way ANOVA

analysis?

In Table 4, now Table 5, the p-values have been double-checked and are correct. We report the

Bonferroni analyses for rheobase, AHP amplitude and AHP time, all of which were shown to be

different by the two-way ANOVA analysis. These post hoc tests did not reveal any further

differences among groups but they were appropriate to run under the circumstances and are

reported.

- Figure 2-4 may have too many colors for data, seeing that data are presented as 3-week-old

vs adult - two colors (for control and PNA) may be enough.

For figures 2-4 we choose to use four colors to differentiate between control and PNA juveniles

and adults because in figure 4, we show example action potential traces from all four groups in

the same four colors. We’ve kept the figure as it was to be consistent throughout the paper.

- Figure 3C: the key for the traces is dots of different colors. These should be lines of different

colors.

Thank you for pointing this out. We have adjusted the key. We have also addressed the next

review comment below. Please see the new figure.

- Figure 3I and J: please give actual p values to match B and K and Figure 2

The p-values have been adjusted to match the rest of the figure and those in figure 2.

- Figure 4A: the current scale is wrong, please revise to show 0 at top and -30 at bottom of

scale.

Thanks for catching this, we have fixed this error.

Reviewer 2:

The authors measured GnRH neuron excitability and action potential properties in control and

postnatally androgenized female mice before puberty and in adulthood using whole-cell current

clamp recording in brain slices. Through a straightforward set of experiments combined with

well described statistical analysis, they showed that development but not prenatally

androgenization is a driver for changes in GnRH neuron intrinsic properties. Their data suggests

that developmentally-induced changes in voltage-gated ion channels in GnRH neurons could

explain the changes in response to synaptic input. Indirectly, it supports mechanisms upstream

of GnRH neurons to explain alterations in spontaneous firing rate after postnatal

androgenization.

Specific comments:

- The abstract contains some inaccuracies: “symptoms resembling PCOS are observed in girls

at or near the time of pubertal onset” What do the authors mean by this affirmation?

A formal diagnosis of PCOS requires two of three criteria: increased androgens,

oligo/anovulation and/or polycystic ovaries. While the first can be documented, cycles are often

irregular near puberty onset and transvaginal ultrasound is not recommended in young girls

because of its invasive nature. We got this phraseology from our clinical colleagues as to how

best characterize what is known about the age of onset of PCOS given these diagnostic

limitations.

We have added these citations to the manuscript. (Line 61)

- Regarding brain slice preparations from adult mice, the day of the estrus cycle on which the

experiment was conducted should be specified.

In the experimental design section, we had stated “gonad-intact control and PNA female mice,

adults studied in diestrus, determined by vaginal cytology.” To provide additional clarification we

added this information to the animals section of the methods: “Adult female mice were studied

on the morning of diestrus, determined via vaginal cytology and uterine mass.” (Lines 95-69)

- The authors nicely document the efficiency of their model (vaginal opening, estrus cyclicity etc)

but data regarding LH levels, LH pulsatility or testosterone levels (even as a citation of previous

study) is missing. In particular, testosterone levels in prepubertal animals would be interesting to

document. Moreover, such PNA model does not always lead to increased androgen levels in

adults.

Reliable testosterone assays require substantial serum not available from prepubertal mice. In

prior work, we attempted to get around this limitation by measuring ovarian content but did not

observe any differences.

Dulka, E. A., Burger, L. L. & Moenter, S. M. Ovarian Androgens Maintain High GnRH Neuron

Firing Rate in Adult Prenatally-Androgenized Female Mice. Endocrinology 161 (2020).

https://doi.org:10.1210/endocr/bqz038

## References

[B1] Abbott DH, Levine JE, Dumesic DA (2016) Translational insight into polycystic ovary syndrome (PCOS) from female monkeys with PCOS-like traits. Curr Pharm Des 22:5625–5633. 10.2174/1381612822666160715133437 27426126 PMC5673087

[B2] Adams C, Stroberg W, DeFazio RA, Schnell S, Moenter SM (2018) Gonadotropin-releasing hormone (GnRH) neuron excitability is regulated by estradiol feedback and kisspeptin. J Neurosci 38:1249–1263. 10.1523/JNEUROSCI.2988-17.2017 29263236 PMC5792479

[B3] Ahn T, Fergani C, Coolen LM, Padmanabhan V, Lehman MN (2015) Prenatal testosterone excess decreases neurokinin 3 receptor immunoreactivity within the arcuate nucleus KNDy cell population. J Neuroendocrinol 27:100–110. 10.1111/jne.12244 25496429 PMC4412353

[B4] Armstrong CM, Matteson DR (1986) The role of calcium ions in the closing of K channels. J Gen Physiol 87:817–832. 10.1085/jgp.87.5.817 2425040 PMC2215889

[B5] Armstrong CM, Hille B (1998) Voltage-gated ion channels and electrical excitability. Neuron 20:371–380. 10.1016/S0896-6273(00)80981-2 9539115

[B6] Berg T, Silveira MA, Moenter SM (2018) Prepubertal development of GABAergic transmission to gonadotropin-releasing hormone (GnRH) neurons and postsynaptic response are altered by prenatal androgenization. J Neurosci 38:2283–2293. 10.1523/JNEUROSCI.2304-17.2018 29374136 PMC5830516

[B7] Bosch MA, Kelly MJ, Rønnekleiv OK (2002) Distribution, neuronal colocalization, and 17beta-E2 modulation of small conductance calcium-activated K(+) channel (SK3) mRNA in the guinea pig brain. Endocrinology 143:1097–1107. 10.1210/endo.143.3.8708 11861537

[B8] Burger LL, Wagenmaker ER, Phumsatitpong C, Olson DP, Moenter SM (2020) Prenatal androgenization alters the development of GnRH neuron and preoptic area RNA transcripts in female mice. Endocrinology 161:bqaa166. 10.1210/endocr/bqaa166 33095238 PMC7583650

[B9] Burt Solorzano CM, McCartney CR, Blank SK, Knudsen KL, Marshall JC (2010) Hyperandrogenaemia in adolescent girls: origins of abnormal gonadotropin-releasing hormone secretion. BJOG 117:143–149. 10.1111/j.1471-0528.2009.02383.x 20002394 PMC2994606

[B10] Burt Solorzano CM, Beller JP, Abshire MY, Collins JS, McCartney CR, Marshall JC (2012) Neuroendocrine dysfunction in polycystic ovary syndrome. Steroids 77:332–337. 10.1016/j.steroids.2011.12.007 22172593 PMC3453528

[B11] Chang RJ, Katz SE (1999) Diagnosis of polycystic ovary syndrome. Endocrinol Metab Clin North Am 28:397–408, vii. 10.1016/S0889-8529(05)70076-1 10352925

[B12] Christian CA, Mobley JL, Moenter SM (2005) Diurnal and estradiol-dependent changes in gonadotropin-releasing hormone neuron firing activity. Proc Natl Acad Sci U S A 102:15682–15687. 10.1073/pnas.0504270102 16230634 PMC1257388

[B13] Chu Z, Tomaiuolo M, Bertram R, Moenter SM (2012) Two types of burst firing in gonadotrophin-releasing hormone neurones. J Neuroendocrinol 24:1065–1077. 10.1111/j.1365-2826.2012.02313.x 22435872 PMC3380170

[B14] Clarke IJ, Cummins JT (1982) The temporal relationship between gonadotropin releasing hormone (GnRH) and luteinizing hormone (LH) secretion in ovariectomized ewes. Endocrinology 111:1737–1739. 10.1210/endo-111-5-1737 6751801

[B15] Coyle C, Campbell RE (2019) Pathological pulses in PCOS. Mol Cell Endocrinol 498:110561. 10.1016/j.mce.2019.110561 31461666

[B16] DeFazio RA, Moenter SM (2002) Estradiol feedback alters potassium currents and firing properties of gonadotropin-releasing hormone neurons. Mol Endocrinol 16:2255–2265. 10.1210/me.2002-0155 12351691

[B17] DeFazio RA, Heger S, Ojeda SR, Moenter SM (2002) Activation of A-type gamma-aminobutyric acid receptors excites gonadotropin-releasing hormone neurons. Mol Endocrinol 16:2872–2891. 10.1210/me.2002-0163 12456806

[B18] DeFazio RA, Navarro MA, Adams CE, Milescu LS, Moenter SM (2019) Estradiol enhances the depolarizing response to GABA and AMPA synaptic conductances in arcuate kisspeptin neurons by diminishing voltage-gated potassium currents. J Neurosci 39:9532–9545. 10.1523/JNEUROSCI.0378-19.2019 31628184 PMC6880461

[B19] Dulka EA, Moenter SM (2017) Prepubertal development of gonadotropin-releasing hormone neuron activity is altered by sex, age, and prenatal androgen exposure. Endocrinology 158:3943–3953. 10.1210/en.2017-00768 28938422 PMC5695838

[B20] Foecking EM, Szabo M, Schwartz NB, Levine JE (2005) Neuroendocrine consequences of prenatal androgen exposure in the female rat: absence of luteinizing hormone surges, suppression of progesterone receptor gene expression, and acceleration of the gonadotropin-releasing hormone pulse generator. Biol Reprod 72:1475–1483. 10.1095/biolreprod.105.039800 15744016

[B21] Franks S (2002) Adult polycystic ovary syndrome begins in childhood. Best Pract Res Clin Endocrinol Metab 16:263–272. 10.1053/beem.2002.0203 12064892

[B22] Gibson AG, Jaime J, Burger LL, Moenter SM (2021) Prenatal androgen treatment does not alter the firing activity of hypothalamic arcuate kisspeptin neurons in female mice. eNeuro 8:ENEURO.0306-21.2021. 10.1523/ENEURO.0306-21.2021 PMC8482853 34503965

[B23] Goodman RL, Hileman SM, Nestor CC, Porter KL, Connors JM, Hardy SL, Millar RP, Cernea M, Coolen LM, Lehman MN (2013) Kisspeptin, neurokinin B, and dynorphin act in the arcuate nucleus to control activity of the GnRH pulse generator in ewes. Endocrinology 154:4259–4269. 10.1210/en.2013-1331 23959940 PMC3800763

[B24] Haisenleder DJ, Dalkin AC, Ortolano GA, Marshall JC, Shupnik MA (1991) A pulsatile gonadotropin-releasing hormone stimulus is required to increase transcription of the gonadotropin subunit genes: evidence for differential regulation of transcription by pulse frequency in vivo. Endocrinology 128:509–517. 10.1210/endo-128-1-509 1702704

[B25] Han SY, McLennan T, Czieselsky K, Herbison AE (2015) Selective optogenetic activation of arcuate kisspeptin neurons generates pulsatile luteinizing hormone secretion. Proc Natl Acad Sci U S A 112:13109–13114. 10.1073/pnas.1512243112 26443858 PMC4620857

[B26] Herbison AE, Moenter SM (2011) Depolarising and hyperpolarising actions of GABA(A) receptor activation on gonadotrophin-releasing hormone neurones: towards an emerging consensus. J Neuroendocrinol 23:557–569. 10.1111/j.1365-2826.2011.02145.x 21518033 PMC3518440

[B27] Hille B (2001) Ion channels of excitable membranes, Ed 3. Sunderland: Sinauer.

[B28] Ibañez L, Potau N, Virdis R, Zampolli M, Terzi C, Gussinyé M, Carrascosa A, Vicens-Calvet E (1993) Postpubertal outcome in girls diagnosed of premature pubarche during childhood: increased frequency of functional ovarian hyperandrogenism. J Clin Endocrinol Metab 76:1599–1603. 10.1210/jcem.76.6.8501168 8501168

[B29] Ibáñez L, Díaz R, López-Bermejo A, Marcos MV (2009) Clinical spectrum of premature pubarche: links to metabolic syndrome and ovarian hyperandrogenism. Rev Endocr Metab Disord 10:63–76. 10.1007/s11154-008-9096-y 18726694

[B30] Knobil E, Neill JD (1994) The physiology of reproduction, Ed 2. New York: Raven Press.

[B31] Legro RS (2003) Diagnostic criteria in polycystic ovary syndrome. Semin Reprod Med 21:267–275. 10.1055/s-2003-43304 14593549

[B32] Levine JE, Norman RL, Gliessman PM, Oyama TT, Bangsberg DR, Spies HG (1985) In vivo gonadotropin-releasing hormone release and serum luteinizing hormone measurements in ovariectomized, estrogen-treated rhesus macaques. Endocrinology 117:711–721. 10.1210/endo-117-2-711 3893989

[B33] Liu X, Herbison AE (2008) Small-conductance calcium-activated potassium channels control excitability and firing dynamics in gonadotropin-releasing hormone (GnRH) neurons. Endocrinology 149:3598–3604. 10.1210/en.2007-1631 18372332 PMC6119466

[B34] Mahoney MM, Padmanabhan V (2010) Developmental programming: impact of fetal exposure to endocrine-disrupting chemicals on gonadotropin-releasing hormone and estrogen receptor mRNA in sheep hypothalamus. Toxicol Appl Pharmacol 247:98–104. 10.1016/j.taap.2010.05.017 20621667 PMC2914852

[B35] McCartney CR, Marshall JC (2016) Polycystic ovary syndrome. N Engl J Med 375:1398–1399. 10.1056/NEJMcp1514916 27705264

[B36] McCartney CR, Eagleson CA, Marshall JC (2002) Regulation of gonadotropin secretion: implications for polycystic ovary syndrome. Semin Reprod Med 20:317–326. 10.1055/s-2002-36706 12536355

[B37] Mendonça PRF, Kyle V, Yeo SH, Colledge WH, Robinson HPC (2018) Kv4.2 channel activity controls intrinsic firing dynamics of arcuate kisspeptin neurons. J Physiol 596:885–899. 10.1113/JP274474 29214635 PMC5830417

[B38] Moenter SM (2015) Leap of faith: does serum luteinizing hormone always accurately reflect central reproductive neuroendocrine activity? Neuroendocrinology 102:256–266. 10.1159/000438790 26278916 PMC4675678

[B39] Moenter SM, Brand RM, Midgley AR, Karsch FJ (1992) Dynamics of gonadotropin-releasing hormone release during a pulse. Endocrinology 130:503–510. 10.1210/endo.130.1.1727719 1727719

[B40] Moore AM, Prescott M, Marshall CJ, Yip SH, Campbell RE (2015) Enhancement of a robust arcuate GABAergic input to gonadotropin-releasing hormone neurons in a model of polycystic ovarian syndrome. Proc Natl Acad Sci U S A 112:596–601. 10.1073/pnas.1415038112 25550522 PMC4299257

[B41] Moore AM, Lohr DB, Coolen LM, Lehman MN (2021) Prenatal androgen exposure alters KNDy neurons and their afferent network in a model of polycystic ovarian syndrome. Endocrinology 162:bqab158. 10.1210/endocr/bqab158 34346492 PMC8402932

[B42] Nagae M, Uenoyama Y, Okamoto S, Tsuchida H, Ikegami K, Goto T, Majarune S, Nakamura S, Sanbo M, Hirabayashi M, Kobayashi K, Inoue N, Tsukamura H (2021) Direct evidence that KNDy neurons maintain gonadotropin pulses and folliculogenesis as the GnRH pulse generator. Proc Natl Acad Sci U S A 118:e2009156118. 33500349 10.1073/pnas.2009156118 PMC7865162

[B43] Naujock M, et al. (2016) 4-Aminopyridine induced activity rescues hypoexcitable motor neurons from amyotrophic lateral sclerosis patient-derived induced pluripotent stem cells. Stem Cells 34:1563–1575. 10.1002/stem.2354 26946488

[B44] Patel K, Coffler MS, Dahan MH, Malcom PJ, Deutsch R, Chang RJ (2004) Relationship of GnRH-stimulated LH release to episodic LH secretion and baseline endocrine-metabolic measures in women with polycystic ovary syndrome. Clin Endocrinol (Oxf) 60:67–74. 10.1111/j.1365-2265.2004.01945.x 14678290

[B45] Pielecka-Fortuna J, DeFazio RA, Moenter SM (2011) Voltage-gated potassium currents are targets of diurnal changes in estradiol feedback regulation and kisspeptin action on gonadotropin-releasing hormone neurons in mice. Biol Reprod 85:987–995. 10.1095/biolreprod.111.093492 21778142 PMC3197916

[B46] Prevot V, Sharif A (2022) The polygamous GnRH neuron: astrocytic and tanycytic communication with a neuroendocrine neuronal population. J Neuroendocrinol 34:e13104. 10.1111/jne.13104 35233849

[B47] Recabarren SE, Recabarren M, Rojas-Garcia PP, Cordero M, Reyes C, Sir-Petermann T (2012) Prenatal exposure to androgen excess increases LH pulse amplitude during postnatal life in male sheep. Horm Metab Res 44:688–693. 10.1055/s-0032-1316291 22763652

[B48] Robinson JE, Birch RA, Foster DL, Padmanabhan V (2002) Prenatal exposure of the ovine fetus to androgens sexually differentiates the steroid feedback mechanisms that control gonadotropin releasing hormone secretion and disrupts ovarian cycles. Arch Sex Behav 31:35–41. 10.1023/a:1014075016956 11910790

[B49] Roland AV, Moenter SM (2011) Prenatal androgenization of female mice programs an increase in firing activity of gonadotropin-releasing hormone (GnRH) neurons that is reversed by metformin treatment in adulthood. Endocrinology 152:618–628. 10.1210/en.2010-0823 21159854 PMC3037157

[B50] Rosenfield RL (2007) Clinical review: identifying children at risk for polycystic ovary syndrome. J Clin Endocrinol Metab 92:787–796. 10.1210/jc.2006-2012 17179197

[B51] Ruka KA, Burger LL, Moenter SM (2016) Both estrogen and androgen modify the response to activation of neurokinin-3 and κ-opioid receptors in arcuate kisspeptin neurons from male mice. Endocrinology 157:752–763. 10.1210/en.2015-1688 26562263 PMC4733114

[B52] Sah P, Faber ES (2002) Channels underlying neuronal calcium-activated potassium currents. Prog Neurobiol 66:345–353. 10.1016/S0301-0082(02)00004-7 12015199

[B53] Shibata R, Nakahira K, Shibasaki K, Wakazono Y, Imoto K, Ikenaka K (2000) A-type K+ current mediated by the Kv4 channel regulates the generation of action potential in developing cerebellar granule cells. J Neurosci 20:4145–4155. 10.1523/JNEUROSCI.20-11-04145.2000 10818150 PMC6772624

[B54] Sidhoum VF, Chan YM, Lippincott MF, Balasubramanian R, Quinton R, Plummer L, Dwyer A, Pitteloud N, Hayes FJ, Hall JE, Martin KA, Boepple PA, Seminara SB (2014) Reversal and relapse of hypogonadotropic hypogonadism: resilience and fragility of the reproductive neuroendocrine system. J Clin Endocrinol Metab 99:861–870. 10.1210/jc.2013-2809 24423288 PMC3942233

[B55] Silva MSB, Desroziers E, Hessler S, Prescott M, Coyle C, Herbison AE, Campbell RE (2019) Activation of arcuate nucleus GABA neurons promotes luteinizing hormone secretion and reproductive dysfunction: implications for polycystic ovary syndrome. EBioMedicine 44:582–596. 10.1016/j.ebiom.2019.05.065 31178425 PMC6606966

[B56] Silveira MA, Burger LL, DeFazio RA, Wagenmaker ER, Moenter SM (2017) GnRH neuron activity and pituitary response in estradiol-induced vs proestrous luteinizing hormone surges in female mice. Endocrinology 158:356–366. 10.1210/en.2016-1771 27911605 PMC5413083

[B57] Simms BA, Zamponi GW (2014) Neuronal voltage-gated calcium channels: structure, function, and dysfunction. Neuron 82:24–45. 10.1016/j.neuron.2014.03.016 24698266

[B58] Simons TJ (1988) Calcium and neuronal function. Neurosurg Rev 11:119–129. 10.1007/BF01794675 2854227

[B59] Storm JF (1987) Action potential repolarization and a fast after-hyperpolarization in rat hippocampal pyramidal cells. J Physiol 385:733–759. 10.1113/jphysiol.1987.sp016517 2443676 PMC1192370

[B60] Sullivan SD, Moenter SM (2004) Prenatal androgens alter GABAergic drive to gonadotropin-releasing hormone neurons: implications for a common fertility disorder. Proc Natl Acad Sci U S A 101:7129–7134. 10.1073/pnas.0308058101 15096602 PMC406477

[B61] Suter KJ, Song WJ, Sampson TL, Wuarin JP, Saunders JT, Dudek FE, Moenter SM (2000) Genetic targeting of green fluorescent protein to gonadotropin-releasing hormone neurons: characterization of whole-cell electrophysiological properties and morphology. Endocrinology 141:412–419. 10.1210/endo.141.1.7279 10614664

[B62] Taylor AE, McCourt B, Martin KA, Anderson EJ, Adams JM, Schoenfeld D, Hall JE (1997) Determinants of abnormal gonadotropin secretion in clinically defined women with polycystic ovary syndrome. J Clin Endocrinol Metab 82:2248–2256. 10.1210/jcem.82.7.4105 9215302

[B63] Tsutsumi R, Webster NJ (2009) GnRH pulsatility, the pituitary response and reproductive dysfunction. Endocr J 56:729–737. 10.1507/endocrj.k09e-185 19609045 PMC4307809

[B64] Veiga-Lopez A, Ye W, Phillips DJ, Herkimer C, Knight PG, Padmanabhan V (2008) Developmental programming: deficits in reproductive hormone dynamics and ovulatory outcomes in prenatal, testosterone-treated sheep. Biol Reprod 78:636–647. 10.1095/biolreprod.107.065904 18094354

[B65] Vickstrom CR, Liu X, Zhang Y, Mu L, Kelly TJ, Yan X, Hu MM, Snarrenberg ST, Liu QS (2020) T-type calcium channels contribute to burst firing in a subpopulation of medial habenula neurons. eNeuro 7:ENEURO.0201-20.2020. 10.1523/ENEURO.0201-20.2020 PMC7433892 32719103

[B66] Vydyanathan A, Wu ZZ, Chen SR, Pan HL (2005) A-type voltage-gated K+ currents influence firing properties of isolectin B4-positive but not isolectin B4-negative primary sensory neurons. J Neurophysiol 93:3401–3409. 10.1152/jn.01267.2004 15647393

[B67] Walters KA, Bertoldo MJ, Handelsman DJ (2018) Evidence from animal models on the pathogenesis of PCOS. Best Pract Res Clin Endocrinol Metab 32:271–281. 10.1016/j.beem.2018.03.008 29779581

[B68] Wildt L, Häusler A, Marshall G, Hutchison JS, Plant TM, Belchetz PE, Knobil E (1981) Frequency and amplitude of gonadotropin-releasing hormone stimulation and gonadotropin secretion in the rhesus monkey. Endocrinology 109:376–385. 10.1210/endo-109-2-376 6788538

[B69] Witham EA, Meadows JD, Shojaei S, Kauffman AS, Mellon PL (2012) Prenatal exposure to low levels of androgen accelerates female puberty onset and reproductive senescence in mice. Endocrinology 153:4522–4532. 10.1210/en.2012-1283 22778229 PMC3423623

[B70] Xiao Y, Barbosa C, Pei Z, Xie W, Strong JA, Zhang JM, Cummins TR (2019) Increased resurgent sodium currents in Nav1.8 contribute to nociceptive sensory neuron hyperexcitability associated with peripheral neuropathies. J Neurosci 39:1539–1550. 10.1523/JNEUROSCI.0468-18.2018 30617209 PMC6381260

[B71] Yan X, Yuan C, Zhao N, Cui Y, Liu J (2014) Prenatal androgen excess enhances stimulation of the GNRH pulse in pubertal female rats. J Endocrinol 222:73–85. 10.1530/JOE-14-0021 24829217

[B72] Yeo SH, Clarkson J, Herbison AE (2014) Kisspeptin-gpr54 signaling at the GnRH neuron is necessary for negative feedback regulation of luteinizing hormone secretion in female mice. Neuroendocrinology 100:191–197. 10.1159/000368608 25301053

